# Development and validation of an LC–MS/MS method for the simultaneous quantification of milbemycin oxime and praziquantel in plasma: application to a pharmacokinetic study in cats

**DOI:** 10.3389/fvets.2023.1285932

**Published:** 2023-10-30

**Authors:** Shiting Xie, Yixing Lu, Jun Wang, Changcheng Lin, Peiyu Ye, Xiaolin Liu, Wenguang Xiong, Zhenling Zeng, Dongping Zeng

**Affiliations:** ^1^Guangdong Provincial Key Laboratory of Veterinary Pharmaceutics Development and Safety Evaluation, College of Veterinary Medicine, South China Agricultural University, Guangzhou, China; ^2^National Risk Assessment Laboratory for Antimicrobial Resistance of Animal Original Bacteria, Guangzhou, China; ^3^Livcare (Guangdong) Animal Health Co., Ltd, Qingyuan, China

**Keywords:** milbemycin oxime, praziquantel, LC–MS/MS, cat plasma, pharmacokinetics

## Abstract

**Introduction:**

Milbemycin oxime (MBO) and praziquantel (PZQ) have a broad spectrum of biological activity and are commonly used to treat the parasitic infection in the veterinary clinic. In this study, a fast and efficient LC-MS/MS method was established and validated for the simultaneous determination of MBO, PZQ, cis-4-hydroxylated-PZQ (C-4-OH-PZQ) and trans-4-hydroxylated-PZQ (T-4-OH-PZQ) and in cat plasma.

**Methods:**

Extraction of analytes and internal standards from cat plasma by acetonitrile protein precipitation, allows rapid processing of large batches of samples. MBO, PZQ, C-4-OH-PZQ, T-4-OH-PZQ, and internal standard (IS) were eluted for 13.5 min on a C_18_ column with a 0.1% formic acid water/acetonitrile mixture as the mobile phase.

**Results:**

Results showed that the method had good precision, accuracy, recovery, and linearity. The linearity range was 2.5–250 ng/mL for MBO, and 10–1000 ng/mL for PZQ, C-4-OH-PZQ, and T-4-OH-PZQ. The intra-day and inter-day precision CV values of the tested components were within 15%. The extraction recoveries of the four components ranged from 98.09% to 107.46%. The analytes in plasma remained stable for 6 h at room temperature, 26 h in the autosampler (4 °C), after freeze–thaw (−20°C) cycles, and 60 days in a −20°C freezer. Method sensitivity sufficed for assessing pharmacokinetic parameters of MBO, PZQ, C-4-OH-PZQ, and T-4-OH-PZQ in plasma samples with LLOQ of 2.5 ng/mL for MBO and 10 ng/mL for PZQ, C-4-OH-PZQ, and T-4-OH-PZQ.

**Conclusion:**

In this study, a selective and sensitive LC-MS/MS method for the simultaneous quantification of MBO, PZQ, C-4-OH-PZQ, and T-4-OH-PZQ in cat plasma was developed and validated.This method had been successfully applied to evaluate the pharmacokinetics of MBO, PZQ, C-4-OH-PZQ, and T-4-OH-PZQ after a single oral administration of 8 mg MBO and 20 mg PZQ in cats.

## 1. Introduction

Praziquantel (PZQ), a broad-spectrum thiazolone-pyrazine-isoquinoline antiparasitic derivative, is safe for the treatment of parasitic infections in cattle and is also used for the treatment of schistosomiasis in humans (1, 2). PZQ is the only drug widely used in schistosomiasis control programs worldwide because it is effective, inexpensive, and easy to administer in one dose (3). PZQ is a racemic mixture composed of equal amounts of the R (−) isomer levo-PZQ and the S (+) isomer dextro-PZQ (4). The R (−) enantiomer has antischistosomal activity, but the (S) enantiomer does not have antischistosomal action (5). R-PZQ is primarily metabolized to R-cis-4-OH-PZQ, whereas S-PZQ is broken down to various mono- and di-hydroxy metabolites in addition to S-trans- and S-cis-4-OH-PZQ (6). PZQ has been used in combination with other various compounds, such as pyrantel pamoate, febantel, and milbemycin oxime, to create broad-spectrum insect repellents for pets (7–9).

Milbemycin oxime (MBO) is a macrocyclic lactone isolated from *Streptomyces*, consisting of nitrile derivatives of 5-didehydromilbemycin, with the proportion of 80% A4 milbemycin (C_32_H_45_NO_7_, MW 555.71) and 20% A3 milbemycin (C_31_H_43_NO_7_, MW541.68) (10). MBO is structurally and chemically similar to avermectin; it exhibits similar anthelmintic, insecticidal, and acaricidal effects at low dose, and is less toxic to mammals (11). MBO is active against the larval and adult stages of intestinal nematodes and the blood stage of heartworm larvae (12). Companion animals may be exposed to infections from internal and external parasites (13). MBO has recently been used in combination with PZQ to develop new treatments against cestodes, nematodes, and ectoparasites in companion animals (14, 15). MBO/PZQ chewable tablets was approved by US-FDA for the prevention of heartworm disease caused by *Dirofilaria immitis* and for the treatment and control of adult roundworm (*Toxocara canis*, *Toxascaris leonina*), adult hookworm (*Ancylostoma caninum*), adult whipworm (*Trichuris vulpis*), and adult tapeworm (*Taenia pisiformis*, *Echinococcus multilocularis*, and *Echinococcus granulosus*) infections (16). The utilization of a combination of MBO and PZQ for cats, exemplified by Milpro® (Virbac, France) and Milbemax® (Novartis, France), has obtained approval (15, 17).

Previous methods have been developed for the analysis of MBO, and PZQ separately (18–20). Few studies have been reported for the simultaneous quantitation of MBO, PZQ, and its main metabolite. The objective of this study was to develop a sensitive, reproducible and efficient LC–MS/MS method to simultaneously quantify MBO, PZQ, and its main metabolite (cis-4-hydroxylated-PZQ and trans-4-hydroxylated-PZQ). The developed method has been fully validated and successfully applied to the pharmacokinetic study of MBO and PZQ receiving oral administration in cats. This method will facilitate the study of bioequivalence.

## 2. Materials and methods

### 2.1. Chemical and standards

Milbemycin oxime (batch number: K0221703, content 98.8%) was purchased from China Veterinary Drug Administration. PZQ (batch number: 100046–202,006, content 99.8%) was purchased from National Institutes for Food and Drug Control. cis-4-hydroxylated-PZQ (C-4-OH-PZQ, batch number: 2-PLL-53-4, content 98.0%), trans-4-hydroxylated- PZQ (T-4-OH-PZQ, batch number: 4-EAW-45-3, content 96.0%), and PZQ-d11 (batch number: 2-ABS-123-3, content 98.0%) were purchased from Toronto Research Chemicals Inc. Moxidectin (batch number: MX-A2207071, content 97.9%) was purchased from Lizhu Group New Beijiang Pharmaceutical Co., Ltd. (China); MBO and PZQ tablets (batch number: 2Q21013, contain 16 mg MBO and 40 mg PZQ) were purchased from Haizheng Animal Health Products Co., Ltd. (Zhejiang, China). Acetonitrile, and methanol of MS grade were obtained from Fisher (Massachusetts, United States). Chromatographic grade formic acid was obtained from Aladdin (Shanghai, China).

### 2.2. Calibration standards and quality control

Stock solutions of MBO, PZQ, C-4-OH-PZQ, T-4-OH-PZQ, moxidectin (IS1), and PZQ-d11(IS2) were prepared in acetonitrile. On the day of analysis, blank plasma was spiked with working solution to prepare calibration and quality control (QC) samples. MBO calibration samples covered a range from 2.5 ng/mL to 250 ng/mL. PZQ, C-4-OH-PZQ, and T-4-OH-PZQ calibration samples covered a range from 10 ng/mL to 1,000 ng/mL. QC samples of 7.5, 30, and 200 ng/mL were used for MBO. QC samples of 30, 120, and 800 ng/mL were used for PZQ, C-4-OH-PZQ, and T-4-OH-PZQ. In each analytical batch, spiked plasma samples (standards and QCs) were extracted with unknown samples.

### 2.3. Sample preparation

A total of 200 μL of cat plasma was extracted with a mixture of 800 μL acetonitrile containing IS. The tube was then agitated on vortex mixer for 30 s ans centrifuged at 12000 rpm for 10 min at 4°C. The 0.5 mL supernatant was added 0.5 mL ultrapure water filtered through a 0.22 μm nylon syringe filter and transferred to microvials for analysis.

### 2.4. Instrument and conditions

The LC–MS/MS system consisted of a Nexera XR HPLC system (Shimadzu, Japan) coupled with an LCMS-8050 triple quadrupole MS (Shimadzu, Japan). LabSolutions 5.99 software was used for data collection and processing. All experiments were performed in positive ion mode using an electrospray ionization (ESI) source. Table 1 shows the MRM transitions and other MS parameters for all analyte in this method.

**Table 1:** Mass spectrometric conditions for each analyte.

Analyte	Ion transition (*m/z*)	CE (V)	RT (min)
MBO-A3	542.2 > 153.1	22	8.1
MBO-A4	556.2 > 167.2	19	8.5
PZQ	313.3 > 203.2	23	4.8
C-4-OH-PZQ	329.1 > 311.1	15	2.7
T-4-OH-PZQ	329.1 > 203.1	15	2.4
PZQ-d11	324.3 > 204.2	23	4.8
Moxidectin	640.3 > 498.2	16	8.6

CE, collision energy; RT, retention time.

The chromatographic column was Gemini C_18_ (50 mm × 2.0 mm; 5 μm; Phenomenex), the solvents were 0.1% formic acid (solvent A) and acetonitrile (solvent B), and the gradient elution rate was 0.3 mL/min. Gradient conditions were optimized as follows: 15–30% B at 0–0.5 min, 30% B at 0.5–2.5 min, 30–50% B at 2.5–2.6 min, 50% B at 2.6–4.5 min, 50–85% B at 4.5–6.0 min, 85% B at 6.0–10.5 min, 85–15% B at 10.5–11.0 min, and 15% B at 11.0–13.5 min. The injection volume was 10 μL. The sample chamber temperature of the autosampler was set to 4°C, and the column temperature was set to 35°C.

### 2.5. Method validation

#### 2.5.1. Linearity

The standard curve samples were prepared according to the protocols described in Section 2.2. The internal standard calibration curves plot the ratio of the analyte response to the internal standard response (response factor) against the ratio of the analyte amount to the internal standard amount. A correlation coefficient (*R*^2^) of 0.99 or above was considered suitable.

#### 2.5.2. Accuracy and precision

Assay accuracy and precision were measured at four different concentrations (2.5, 7.5, 30, and 200 ng/mL of MBO; 10, 30, 120, and 800 ng/mL of PZQ, C-4-OH-PZQ, and T-4-OH-PZQ). Accuracy and precision were determined between runs (intra-assay) and recorded on three different days (inter-assay). The precision of the method was assessed using the coefficient of variation (CV), with a CV ≤ 15% being an acceptable value. Accuracy was expressed as a percentage of the measured QC concentration relative to the nominal value.

#### 2.5.3. Recovery and matrix effect

Absolute recoveries of MBO, PZQ, C-4-OH-PZQ, and T-4-OH-PZQ were assessed using the calculated ratio of blank plasma; the concentration spiked into QC samples to its nominal concentration. Matrix effects were assessed by comparing the peak areas of the extracted samples with those of pure solutions of the same concentration.

#### 2.5.4. Stability

To assess stability, QC plasma samples of MBO (7.5 and 200.0 ng/mL), PZQ (30.0 and 80.0 ng/mL), C-4-OH-PZQ (30.0 and 800.0 ng/mL), and T-4-OH-PZQ (30.0 and 800.0 ng/mL) were subjected to short-term (6 h) incubation at room temperature, long-term (60 d) in −20°C, three freeze–thaw (−20°C) cycles, and 26 h in the autosampler (4°C). Subsequently, MBO, PZQ, C-4-OH-PZQ, and T-4-OH-PZQ concentrations were measured and compared with freshly prepared samples.

### 2.6. Pharmacokinetics

All animal procedures were approved by the Institutional Animal Care and Use Committee of South China Agricultural University (approval number: 2023A011), and the cats were handled with due regard for their welfare and in compliance with all local and national regulatory requirements. Six male cats were studied to determine the PK profiles of MBO, PZQ and its main metabolite (C-4-OH-PZQ and T-4-OH-PZQ). The cats were ≥ 12 months of age and weighed between 2.7 and 3.8 kg. The cats were fasted overnight and allowed free access to water prior to PK study. A single dose of 8 mg MBO and 20 mg PZQ formulation was administered to cats. 1 mL of blood samples were drawn from the antecubital vein and collected in tubes containing heparin at the following times: 0, 0.16, 0.5, 0.75,1, 2,3, 4, 6, 9, 12, 16, 24, 36, 48, 72, 120, 168, and 216 h. Blood samples were centrifuged at 3500 rpm for 10 min at 4°C to separate plasma and stored at −20°C until analysis. The plasma PK parameters of MBO, PZQ, C-4-OH-PZQ, and T-4-OH-PZQ were determined by non-compartmental analysis in Phoenix WinNonlin® 8.2 (Certara, L.P., Princeton, NJ, United States).

## 3. Results and discussion

### 3.1. LC–MS/MS method development

**Figure 1:**
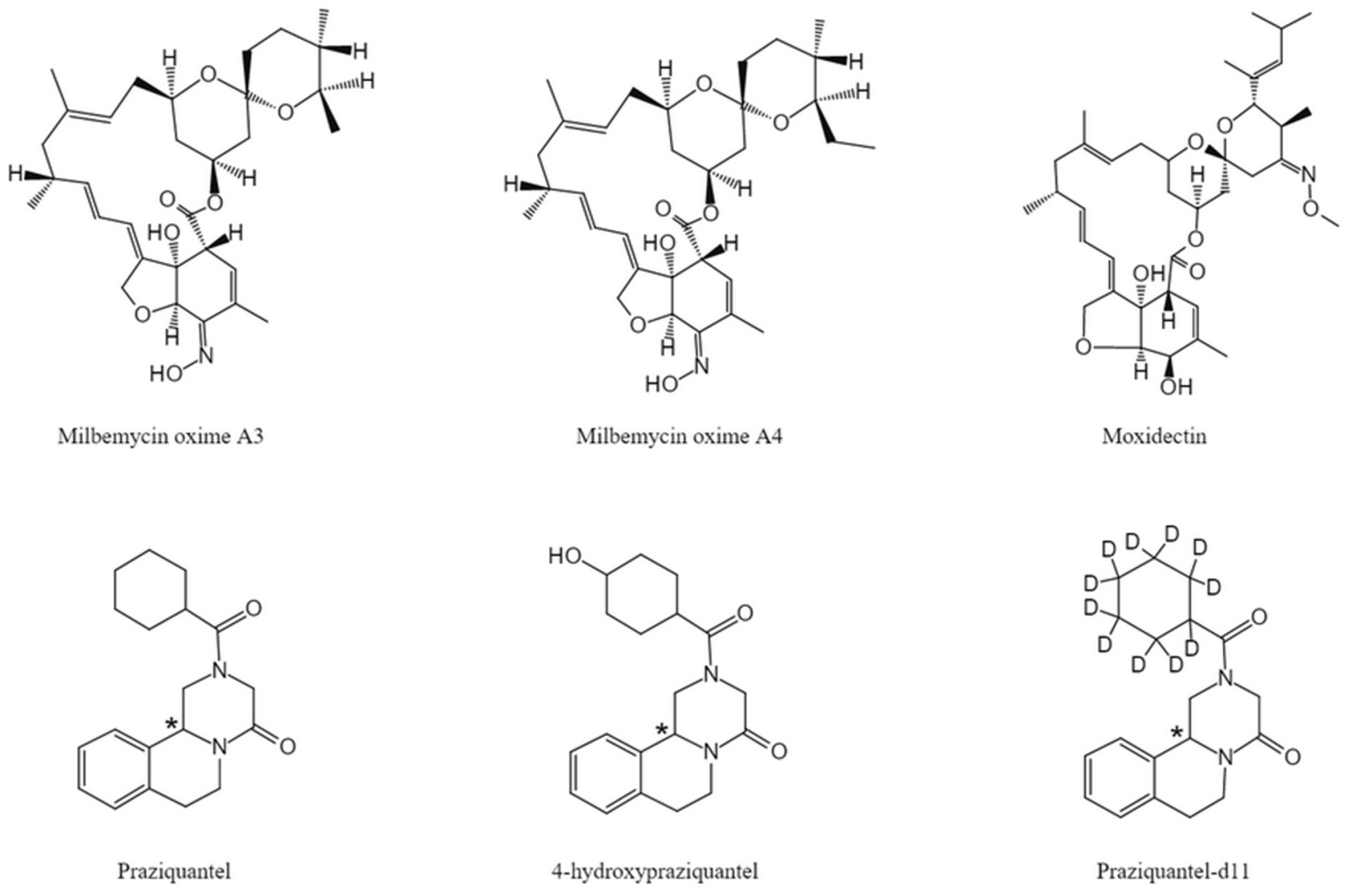
Chemical structures of MBO-A3, MBO-A4, moxidectin (IS), PZQ, 4-OH-PZQ, PZQ-d11 (IS). *The chiral center.

The chemical structures of MBO, PZQ, 4-OH-PZQ, moxidectin (IS1) and PZQ-d11 (IS2) are shown in Figure 1. MBO-A3, MBO-A4, PZQ, C-4-OH-PZQ, T-4-OH-PZQ, IS1, and IS2 were protonated in the positive ESI mode, and [M + H] ^+^ at *m/z* 542.2, 556.2, 313.3, 329.1, 329.1, 640.3 and 324.3 was selected as the precursor ion. The selected product fragment ions *m/z* were 153.1, 167.2, 203.2, 311.1, 203.1,498.2 and 204.2 for MBO-A3, MBO-A4, PZQ, C-4-OH-PZQ, T-4-OH-PZQ, IS1, and IS2, respectively. Different LC conditions were tested to obtain suitable retention time and separation MBO-A3, MBO-A4, PZQ, C-4-OH-PZQ, T-4-OH-PZQ, IS1, and IS2. To avoid cross-peaks, gradient elution was used to separate the different analytes on a C_18_ column. Methanol, acetonitrile, 5 mM ammonia acetate (pH = 7.6), 0.1% formic acid, and 100% water were used as potential mobile phases. A gradient elution was established as described in section 2.4. The results showed that when eluted with 0.1% formic acid and acetonitrile, the peak resolution and peak shape are better. Under the optimized conditions, MBO-A3, MBO-A4, PZQ, C-4-OH-PZQ, T-4-OH-PZQ, IS1, and IS2 were separated on a C_18_ column eluted with 0.1% formic acid and acetonitrile under gradient conditions (13.5 min). These conditions allowed for high chromatographic resolution with clear MBO-A3, MBO-A4, PZQ, C-4-OH-PZQ, T-4-OH-PZQ, IS1 and IS2 peaks at 8.1, 8.5, 4.8, 2.7, 2.4, 8.6, and 4.8 min, respectively.

### 3.2. Selection of IS

The use of internal standards can substantially reduce the random errors caused by the extraction process, ionization, and injection (21). To obtain better accuracy and precision, abamectin and moxidectin were tested as IS for MBO. Due to the instability of abamectin, moxidectin was finally selected as IS for MBO because it not only reduces the influence of the matrix on the analyte, but also more effectively reduced in the influence of the volume changes during the sample extraction. PZQ-d11 was finally selected as IS for PZQ, C-4-OH-PZQ, and T-4-OH-PZQ (22).

### 3.3. Sample preparation

Some of the most commonly used sample preparation methods for the simultaneous purification of plasma samples and the extraction of drugs include protein precipitation (23), liquid–liquid extraction (24), solid phase extraction (25), and their combinations (26). In this study, the one-step protein precipitation method was ultimately selected given its rapid and simple performance. During method development, we used methanol and acetonitrile as solvents for protein precipitation, and ultimately selected acetonitrile because of its extended recovery yield. The pretreatment method can analyze a large number of plasma samples in a short time.

### 3.4. Method validation

#### 3.4.1. Selectivity

**Figure 2:**
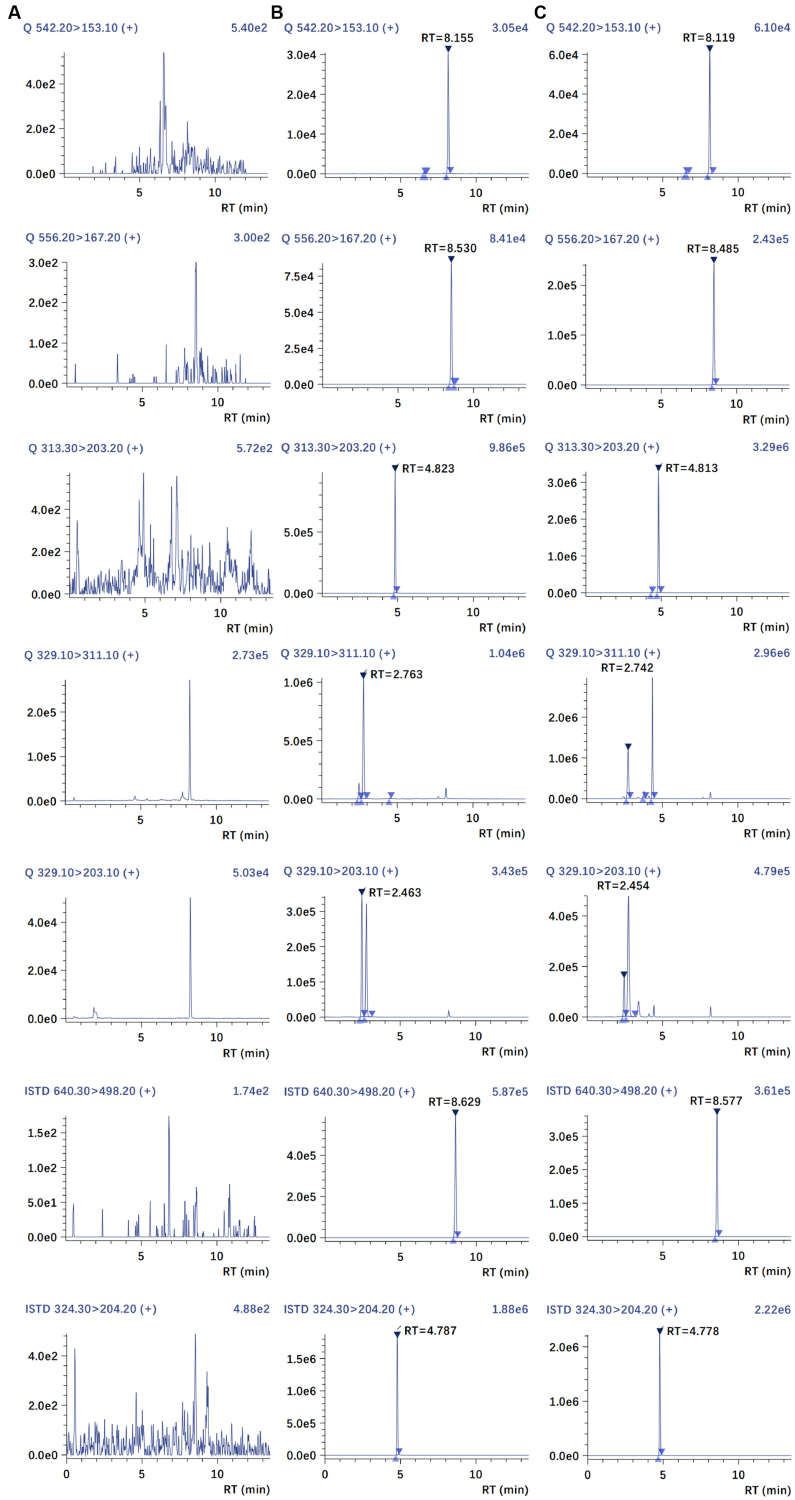
Representative MRM chromatograms of blank plasma **(A)**, blank plasma spiked with MBO, PZQ, C-4-OH-PZQ, T-4-OH-PZQ, moxidectin (IS1), and PZQ-d11(IS2) **(B)**, and a PK sample at 3 h **(C).**

The chromatograms of blank plasma, blank plasma spiked with MBO, PZQ, C-4-OH-PZQ, T-4-OH-PZQ, IS1, and IS2, and a PK sample at 3 h are shown in Figure 2. Comparing these chromatograms, no endogenous interferences were detected in the retention times of the analyte and IS. Therefore, the plasma background does not interfere with MBO, PZQ, C-4-OH-PZQ, T-4-OH-PZQ and IS, suggesting the selectivity and specificity of the MRM.

#### 3.4.2. Linearity

The calibration standards corrected by IS were prepared from MBO, PZQ, C-4-OH-PZQ, and T-4-OH-PZQ standards, where IS (2000 ng/mL moxidectin and 200 ng/L PZQ-d11) spiked for each concentration. Data were analyzed using weighted least squares linear regression with a weighting factor of 1/x^2^. The developed method was linear in the range of 2.5–250 ng/mL for MBO and 10–1,000 ng/mL for PZQ, C-4-OH-PZQ, and T-4-OH-PZQ (Table 2). Calibration curve *R*^2^ value ≥0.998. The LLOQ was 2.5 ng/mL for MBO and 10 ng/mL for PZQ, C-4-OH-PZQ, and T-4-OH-PZQ with a signal-to-noise (S/N) ratio > 10. The LLOQ of PZQ in this method has lower than previously reported methods (27).

**Table 2:** Line ranges for MBO, PZQ, C-4-OH-PZQ, and T-4-OH-PZQ in cat plasma.

Analyte	LLOQ (ng/mL)	Line range (ng/mL)	Linear equation in plasma	*R* ^2^
MBO	2.5	2.5–250	Y = 1.26393X + 0.00594	0.999
PZQ	10	10–1,000	Y = 0.07010X + 0.00382	0.998
C-4-OH-PZQ	10	10–1,000	Y = 0.15114X + 0.00496	0.999
T-4-OH-PZQ	10	10–1,000	Y = 0.04648X + 0.00216	0.999

#### 3.4.3. Accuracy and precision

Table 3 shows the intraday and interday precision and accuracy of MBO, PZQ, C-4-OH-PZQ, and T-4-OH-PZQ. The intraday precision of the method for determining MBO was 1.69–8.34%, with accuracies ranging from 98.39–105.18%. The intraday precisions of PZQ, C-4-OH-PZQ, and T-4-OH-PZQ were 2.50–5.35%, 2.76–3.72%, and 3.31–3.75%, with accuracies of 97.90–99.34%, 100.97–106.79%, and 98.97–108.09%. The interday precision was 4.54–9.98%, with accuracy of 91.78–101.33% for MBO. The interday precisions were 3.66–4.64%, 4.60–7.63%, and 4.57–7.13%, with accuracies of 96.29–101.02%, 96.13–102.69%, and 92.00–103.80% for measuring PZQ, C-4-OH-PZQ, and T-4-OH-PZQ concentrations, respectively.

**Table 3:** Precision and accuracy of MBO, PZQ, C-4-OH-PZQ, and T-4-OH-PZQ in cat plasma by LC–MS/MS.

Analyte	Nominal concentration (ng/mL)	Intra-day (*n* = 6)			Inter-day (*n* = 3 × 6)		
		Measured concentration (mean ± SD, ng/mL)	Precision (CV%)	Accuracy (%)	Measured concentration (mean ± SD, ng/mL)	Precision (CV%)	Accuracy (%)
MBO	2.5	2.46 ± 0.21	8.34	98.39	2.28 ± 0.23	9.98	91.78
	7.5	7.89 ± 0.49	6.14	105.18	7.57 ± 0.46	6.13	100.88
	30	31.47 ± 1.42	4.51	104.91	30.14 ± 1.37	4.54	100.45
	200	207.25 ± 3.51	1.69	103.63	202.66 ± 10.69	5.27	101.33
PZQ	10	9.84 ± 0.53	5.35	98.39	10.10 ± 0.45	4.46	101.02
	30	29.37 ± 0.97	3.29	97.90	30.01 ± 1.17	3.90	100.04
	120	119.21 ± 4.63	3.88	99.34	115.54 ± 5.36	4.64	96.29
	800	791.62 ± 19.77	2.50	98.95	771.04 ± 28.18	3.66	96.38
C-4-OH-PZQ	10	10.64 ± 0.29	2.76	106.42	10.27 ± 0.78	7.63	102.69
	30	32.04 ± 1.19	3.72	106.79	29.96 ± 1.98	6.61	99.87
	120	121.16 ± 3.91	3.22	100.97	116.97 ± 5.69	4.86	97.47
	800	809.46 ± 24.52	3.03	101.18	769.00 ± 35.35	4.60	96.13
T-4-OH-PZQ	10	9.90 ± 0.37	3.75	98.97	9.34 ± 0.67	7.13	92.00
	30	32.43 ± 1.21	3.74	108.09	30.43 ± 1.97	6.47	101.43
	120	125.95 ± 4.17	3.31	104.96	124.36 ± 5.69	4.57	103.80
	800	847.12 ± 28.71	3.39	105.89	828.30 ± 49.59	5.99	103.54

#### 3.4.4. Recovery and matrix effect

Table 4 shows the evaluation results of matrix effects and recoveries. After pretreatment by this method, the mean extraction recovery of MBO in cat plasma at three QC concentrations (7.5, 30, and 200 ng/mL) were 96.91–100.62%. The mean extraction recoveries at 30, 120, and 800 μg/mL were 100.97–104.90%, 103.03–107.46%, and 104.91–106.97% for PZQ, C-4-OH-PZQ, and T-4-OH-PZQ, respectively. The average extraction yields of IS1 (2000 ng/mL) and IS2 (200 ng/mL) were 98.06 and 103.68%, respectively. The CV (%) value of the estimated extraction rate was within ±15%, indicating a high reproducibility of the sample preparation process.

**Table 4:** Recovery and matrix effect of MBO, PZQ, C-4-OH-PZQ, and T-4-OH-PZQ (*n* = 6).

Analyte	Nominal concentration (ng/mL)	Recovery (%)		Matrix Effect (%)	
		Mean ± SD (%)	CV (%)	Mean ± SD (%)	CV (%)
MBO	7.5	98.09 ± 5.47	5.58	80.28 ± 10.34	12.88
	30	100.62 ± 3.56	3.54	91.03 ± 4.29	4.71
	250	96.91 ± 1.87	1.93	82.96 ± 6.62	7.70
	Mean	98.54 ± 4.01	4.07	85.76 ± 10.16	11.85
PZQ	30	102.53 ± 7.76	7.57	100.68 ± 11.29	11.22
	120	104.90 ± 6.81	6.49	97.37 ± 8.11	8.33
	800	100.97 ± 10.01	9.91	99.65 ± 6.12	6.14
	Mean	102.80 ± 7.97	7.76	99.23 ± 8.36	8.42
C-4-OH-PZQ	30	107.46 ± 2.83	2.64	95.71 ± 3.55	3.71
	120	103.03 ± 1.42	1.38	98.39 ± 3.46	3.51
	800	105.64 ± 2.16	2.05	96.38 ± 3.21	3.33
	Mean	105.38 ± 2.80	2.66	96.83 ± 3.41	3.52
T-4-OH-PZQ	30	106.97 ± 2.69	2.52	105.57 ± 3.49	3.30
	120	104.91 ± 1.52	1.45	108.71 ± 4.21	3.88
	800	105.92 ± 3.35	3.16	104.74 ± 2.08	1.99
	Mean	105.93 ± 2.62	2.47	106.34 ± 3.63	3.41
Moxidecin	2000	98.06 ± 3.56	3.63	82.49 ± 8.94	10.84
PZQ-d11	200	103.68 ± 5.42	5.22	105.77 ± 6.42	6.07

The mean matrix effects for MBO, PZQ, C-4-OH-PZQ, and T-4-OH-PZQ at QC concentrations were 80.28–91.03%, 97.37–100.68%, 95.71–98.39%, and 104.74–108.71%, respectively. For IS1 (2000 ng/mL) and IS2 (200 ng/mL), the average matrix effects were 82.49 and 105.77%, respectively. The CV (%) value of the matrix effect was within ±15%. After normalization with IS, the matrix effects for MBO, PZQ, C-4-OH-PZQ, and T-4-OH-PZQ in cat plasma were 102.87–105.31%, 90.34–97.67%, 86.31–94.74%, and 95.19–104.67%, respectively. No significant matrix effect was observed under experimental conditions.

#### 3.4.5. Plasma stability

Stability tests showed that MBO, PZQ, C-4-OH-PZQ, and T-4-OH-PZQ were stable in three freeze–thaw cycles, for 6 h at room temperature, after processing for 26 h at 4°C, and storage (−20°C) condition (Table 5). Four stability tests obtained an accuracy between 93.00 and 111.07% and an RSD% ≤10% for all analytes. All analytes were considered storage stable according to FDA criteria (28) with an accuracy of ±15% at each level under all conditions tested.

**Table 5:** Stability tests for MBO, PZQ, C-4-OH-PZQ, and T-4-OH-PZQ in cat plasma (*n* = 6).

Analyte	Nominal concentration (ng/mL)	25°C for 6 h		Freeze–Thaw Stability (3 Cycles)		Autosampler (26 h, 4°C)		Long-term (−20°C, 45 Days)		Long-term (−20°C, 60 Days)	
		Measured concentration (ng/mL)	Accuracy (%)	Measured concentration (ng/mL)	Accuracy (%)	Measured concentration (ng/mL)	Accuracy (%)	Measured concentration (ng/mL)	Accuracy (%)	Measured concentration (ng/mL)	Accuracy (%)
MBO	7.5	7.68	102.40	7.61	101.47	7.48	99.73	7.83	104.40	8.33	111.07
	200	203.56	101.78	198.23	99.12	192.42	96.21	217.49	108.75	207.55	103.78
PZQ	30	31.05	103.50	30.88	102.93	31.03	103.43	27.90	93.00	28.72	95.73
	800	769.29	96.16	766.45	95.81	790.78	98.85	768.32	96.04	773.52	96.69
C-4-OH-PZQ	30	30.46	101.53	29.04	96.80	31.63	105.43	29.93	99.77	31.53	105.10
	800	814.00	101.75	790.93	98.87	785.35	98.17	791.45	98.93	815.35	101.92
T-4-OH-PZQ	30	30.68	102.27	29.44	98.13	30.65	102.17	28.62	95.40	28.94	96.47
	800	811.74	101.47	791.44	98.93	773.09	96.64	747.69	93.46	746.74	93.34

#### 3.4.6. Dilution integrity

When plasma sample concentrations exceed the ULOQ, a five-fold dilution was performed to quantify MBO, PZQ, C-4-OH-PZQ, and T-4-OH-PZQ in the cat plasma samples within the range of the standard curve. Analytes were diluted five-fold with empty cat plasma to QC concentrations of 200 ng/mL (MBO) and 800 ng/mL (PZQ, C-4-OH-PZQ, and T-4-OH-PZQ), and plasma samples were collected in duplicate six times to check the completeness of the dilution. Table 6 represents the results of the five-fold dilution validation experiment of MBO, PZQ, C-4-OH-PZQ, and T-4-OH-PZQ in cat plasma. The accuracy and precision of the diluted concentrations satisfied the acceptance criteria, defined as ±15% of the nominal concentrations, indicating that the bioanalytical method is valid for dilution integrity of samples at concentrations exceeding the ULOQ.

**Table 6:** five-fold dilution integrity of MBO, PZQ, C-4-OH-PZQ, and T-4-OH-PZQ in cat plasma (*n* = 6).

Analyte	Nominal concentration (ng/mL)	Dilution factor 5		
		Mean ± SD (%)	CV (%)	Accuracy (%)
MBO	200	218.02 ± 2.43	1.58	109.01
PZQ	800	794.13 ± 38.06	4.79	99.27
C-4-OH-PZQ	800	803.58 ± 37.12	4.62	100.45
T-4-OH-PZQ	800	835.77 ± 37.33	4.47	104.47

### 3.5. Application in a PK study is cats

**Figure 3:**
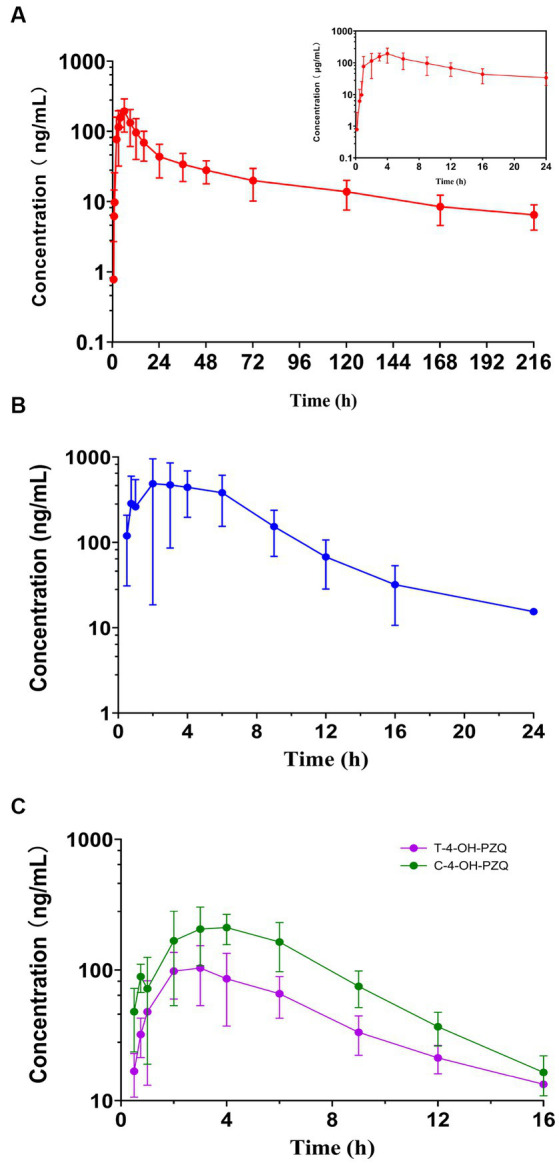
Plasma concentration–time profile of MBO **(A)**, PZQ **(B)**, C-4-OH-PZQ and T-4-OH-PZQ **(C)** in cats following oral administration of 8 mg MBO and 20 mg PZQ (mean ± SD, *n* = 6).

The mean MBO, PZQ, C-4-OH-PZQ, and T-4-OH-PZQ plasma concentration–time profiles after oral administration of 8 mg MBO and 20 mg PZQ to cats (*n* = 6) are shown in Figure 3. The PK parameters are listed in Table 7. The mean AUC_0 − t_ values of MBO, PZQ, C-4-OH-PZQ, and T-4-OH-PZQ were 4820.76, 3593.22, 1521.50, and 664.87 ng·h/mL following oral administration of 8 mg MBO and 20 mg PZQ. The PZQ, C-4-OH-PZQ, and T-4-OH-PZQ were rapidly eliminated, with mean CL/*F* values of 2.08, 3.89, and 8.55 mL/h/kg and *t_1/2_* of 2.71, 3.19, and 4.72 h after oral administration of 20 mg PZQ. The MBO was slowly eliminated, with mean CL/F value of 0.54 mL/h/kg and *t_1/2_* of 49.91 h after oral administration of 8 mg MBO. The validated method was successfully applied in a PK study for simultaneous quantification of MBO, PZQ and its main metabolite in cats. Furthermore, this method can be applied to a bioequivalence study.

**Table 7:** Pharmacokinetic parameters of 8 mg MBO and 20 mg PZQ after oral administration to cats (*n* = 6).

Parameters	MBO	PZQ	C-4-OH-PZQ	T-4-OH-PZQ
*t_1/2_* (h)	49.91 ± 22.71	2.71 ± 0.80	3.19 ± 0.73	4.72 ± 2.67
T_max_ (h)	4.33 ± 1.37	3.67 ± 1.86	4.00 ± 1.67	3.83 ± 1.83
C_max_ (ng/mL)	251.36 ± 59.27	637.54 ± 351.34	262.69 ± 36.19	117.48 ± 36.69
AUC_0-t_ (ng·h/mL)	4820.76 ± 2054.46	3593.22 ± 2079.93	1521.50 ± 156.60	664.87 ± 183.26
AUC_0-∞_ (ng·h/mL)	5267.86 ± 2199.14	3701.29 ± 2080.69	1616.80 ± 190.09	768.83 ± 214.41
Vz/F (L/kg)	33.97 ± 10.81	8.20 ± 5.50	17.64 ± 4.03	51.95 ± 14.72
CL/F (L/h/kg)	0.54 ± 0.25	2.08 ± 1.03	3.89 ± 0.72	8.55 ± 2.08

PZQ is the drug of choice for the treatment of schistosomiasis and is widely used in preventive chemotherapy programs (as defined by WHO) (29). PZQ is metabolized by multiple CYPs, and so other drugs within these CYP pathways may lead to the formation and accumulation of metabolic by-products or a reduce the drug’s therapeutic efficacy of the drug (30). Although the structure and properties of MBO are very close to ivermectin, it is safer and has fewer side effects (31). There are a variety of compound antiparasitic products for pets. The impact of different combination of antiparasitic drugs on their respective pharmacokinetics requires further study (32). The LC–MS/MS method established in this study provides a practical means for the quantitative analysis of MBO and PZQ and metabolites. In addition, this method can also be applied to a bioequivalence studies.

## 4. Conclusion

In this study, a selective and sensitive LC–MS/MS method for the simultaneous quantification of MBO, PZQ, C-4-OH-PZQ, and T-4-OH-PZQ in cat plasma was developed and validated. The short run time (13.5 min) simplicity and reproducibility of the extraction method are valuable advantages for the analysis of a large number of samples. The sensitivity in plasma was 2.5 ng/mL for MBO and 10 ng/mL for PZQ, C-4-OH-PZQ, and T-4-OH-PZQ. The applicability of this method was demonstrated by analyzing the PK profile of cats after a single oral dose of 8 mg MBO and 20 mg PZQ.

## Data availability statement

The original contributions presented in the study are included in the article/supplementary material, further inquiries can be directed to the corresponding authors.

## Ethics statement

The animal study was approved by Institutional Animal Care and Use Committee of South China Agricultural University. The study was conducted in accordance with the local legislation and institutional requirements.

## Author contributions

SX: Conceptualization, Formal analysis, Investigation, Methodology, Validation, Writing – original draft, Writing – review & editing. YL: Formal analysis, Methodology, Validation, Visualization, Writing – original draft, Writing – review & editing. JW: Formal analysis, Investigation, Methodology, Writing – original draft. CL: Formal analysis, Investigation, Methodology, Writing – original draft. PY: Formal analysis, Methodology, Writing – original draft. XL: Formal analysis, Investigation, Methodology, Writing – original draft. WX: Conceptualization, Formal analysis, Writing – original draft. ZZ: Conceptualization, Funding acquisition, Project administration, Writing – review & editing, Writing – original draft. DZ: Conceptualization, Project administration, Writing – review & editing, Writing – original draft.
